# Supplementary material for: Systemic and Mucosal Humoral Immune Responses to Lumazine Synthase 60-mer Nanoparticle SARS-CoV-2 Vaccines
Source: Vaccines (Basel). 2025 Jul 23;13(8):780. doi: 10.3390/vaccines13080780 (PMC12390229; doi:10.3390/vaccines13080780)
Supplement: Supplementary file 1 [file vaccines-13-00780-s001.zip › vaccines-3676184-supplementary.pdf]

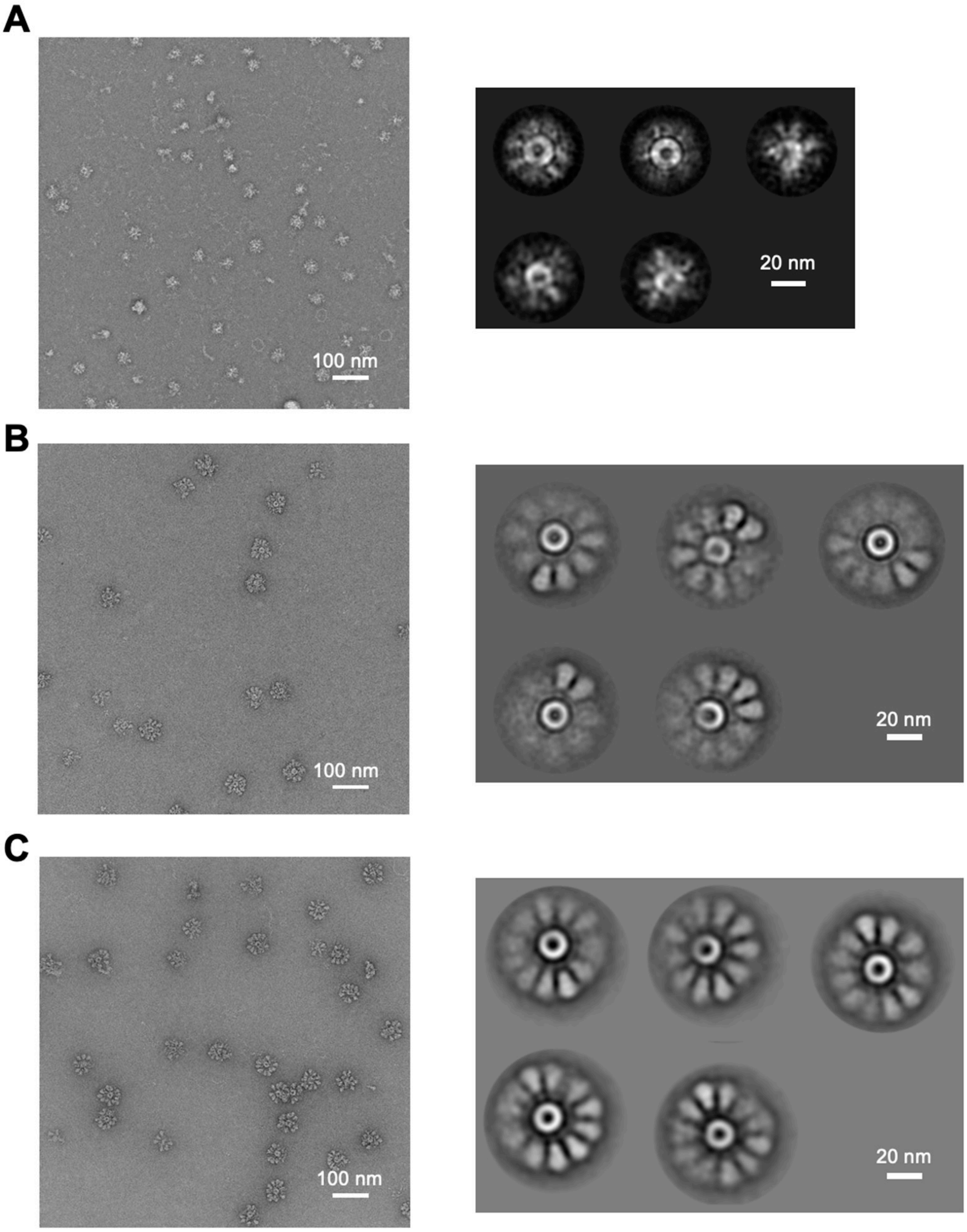

Supplementary Figure S1 associated with Figure 1. Negative-stain electron microscopy of nanoparticles. Representative raw micrographs (left) and 2D class averages (right) of S-2P-LuS (**A**), S-6P-LuS14 parental (**B**) and S-6P-LuS14 Beta variant (B.1.351) (**C**).

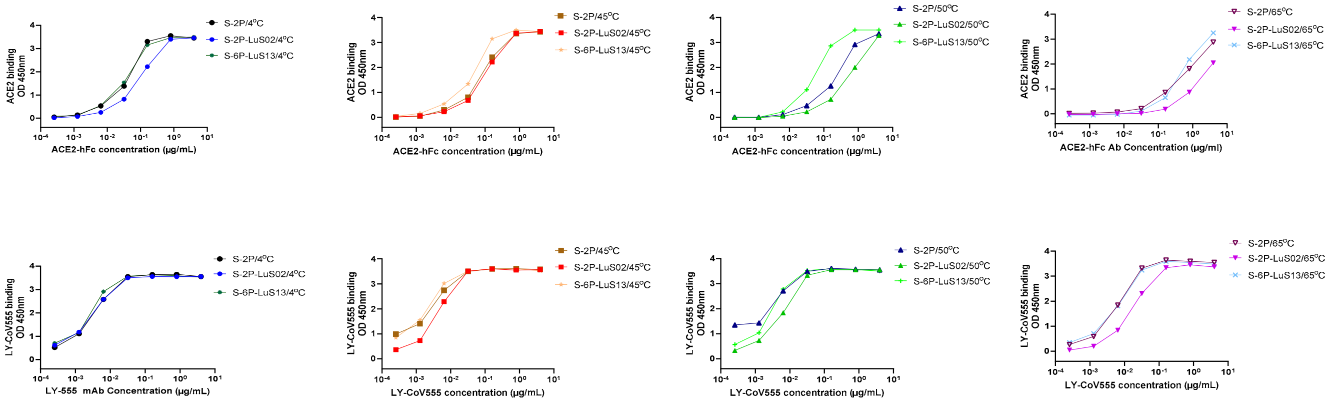

Supplementary Figure S2 associated with Figure 2. Stability of S-2P trimer and nanoparticle vaccines when heated. Purified immunogens were heated at temperatures ranging from 4 °C to 65 °C for 10 minutes and then coated on ELISA plates to measure their binding to ACE2-hFc or anti-COVID neutralizing antibody LY-CoV555.

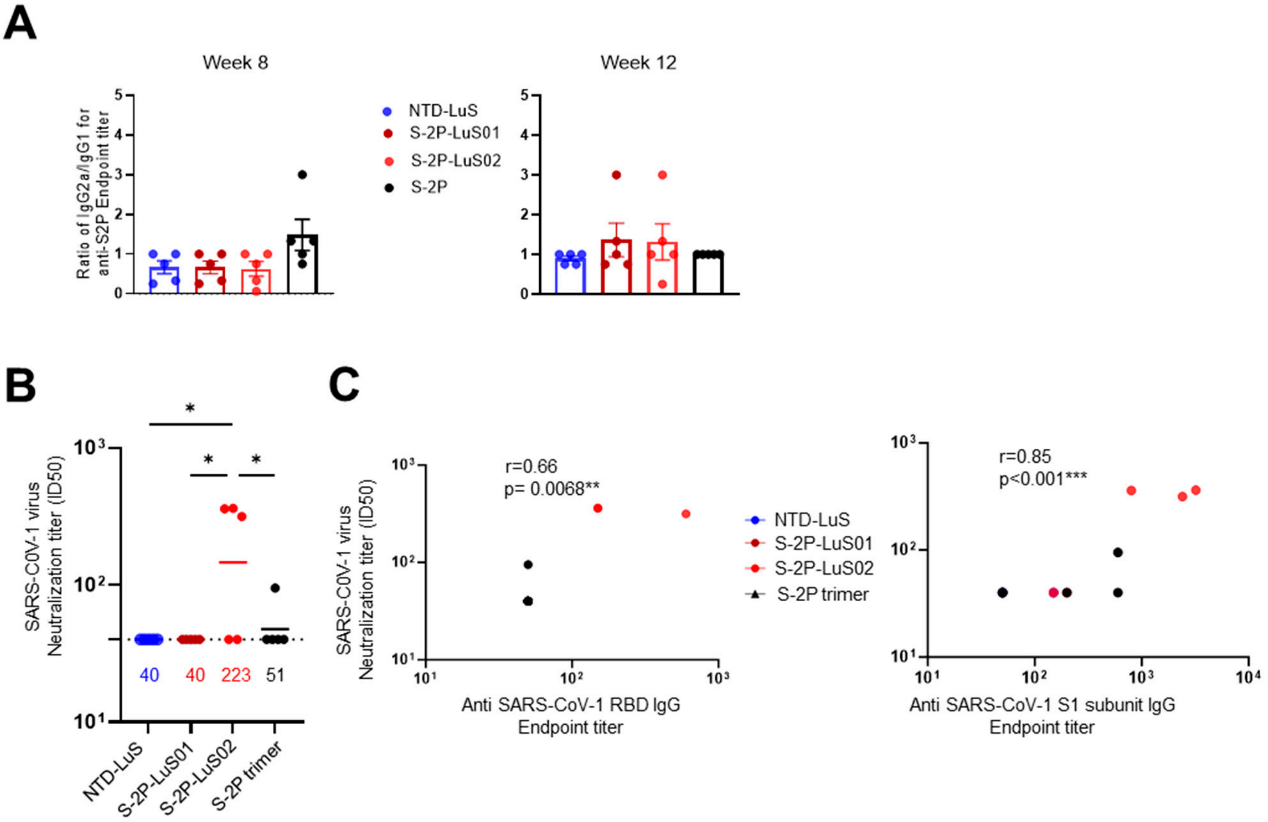

Supplementary Figure S3 associated with Figure 4. Anti-SARS CoV-2 S-2P IgG2a/IgG1 ratio and cross-reactive neutralization activity against SARS-CoV-1 post two immunizations. (A). A balanced Th1 and Th2 immune response as measured with ratio of IgG2a/IgG1 for anti-S-2P IgG titers post two immunizations at week 8 and 12. (B) Serum neutralization titer (ID50) from pseudovirus neutralization assay against SARS-CoV-1 at week 5. (C) Correlation of SARS-CoV-1 ID50 titer with anti-SARS-CoV-1 RBD or anti-SARS-CoV-1 S1 subunit ELISA titers. Correlation analysis at week 5 was performed using two-tailed Pearson correlation coefficient test.
